# Prediction of Microvascular Adaptation to Hypoxia Based on Myogenic Microcirculation Oscillations

**DOI:** 10.3390/s25092751

**Published:** 2025-04-26

**Authors:** Andrzej Marcinek, Joanna Katarzynska, Artur Stanek, Jerzy Gebicki

**Affiliations:** 1 Institute of Applied Radiation Chemistry, Lodz University of Technology, 90-924 Lodz, Poland; 2 Angionica Ltd., 90-924 Lodz, Poland; joanna.katarzynska@angionica.com.pl; 3 Hypoxico Polska Ltd., 42-202 Czestochowa, Poland; a.stanek@hypoxico.com.pl

**Keywords:** microcirculation, myogenic oscillations, hypoxia sensitivity, NADH fluorescence, FMSF technique

## Abstract

Microcirculatory oscillations known as flowmotion are a recognized feature of blood flow that reflect the functional state of the vascular system. Many diseases are associated with impaired flowmotion, especially diseases that are accompanied by hypoxia. Low-frequency myogenic oscillations (0.052–0.15 Hz) are an important regulator of microvascular adaptation to hypoxia. Here, we study the myogenic component of flowmotion using the FMSF–PORH (Flow Mediated Skin Fluorescence–Post Occlusive Reactive Hyperemia) technique. Myogenic oscillations were strongly activated under hypoxic conditions caused by occlusion of the brachial artery or intermittent hypoxic treatment. A strong correlation was noted between the hypoxia sensitivity parameter HS (the intensity of myogenic oscillations activated by hypoxia) and the normoxic myogenic flowmotion parameter VM (the intensity of myogenic oscillations under normoxic conditions). If HS is considered as a direct measure of the microcirculation response to hypoxia, then VM can be considered a measure of the microcirculation’s readiness to provide this response. The predictive value of the VM parameter is presented. The assessment of myogenic activity under normoxia conditions could thus provide a simple and rapid diagnostic tool for health care practitioners.

## 1. Introduction

Microcirculatory blood flow oscillations known as flowmotion are a direct result of vasomotion—i.e., fluctuations in vessel diameter, synchronous dilation of vessels or constriction over the time. Vasomotion quality differs between vascular beds and is of significant importance for microcirculation, especially under pathological conditions when blood supply to the organ is impaired. The mechanistic aspects of flowmotion, especially its role in ensuring the proper perfusion of tissues, have been the subject of extensive research [1,2,3,4,5,6,7,8,9,10].

Microcirculation is mostly studied in humans at the level of the skin, partly because skin remains the most accessible measurement site. More importantly, however, skin microcirculation dysfunction mirrors impairment of microcirculation and blood flow oscillations in other organs. The majority of data on flowmotion are collected via measurements of blood flow (laser-Doppler flowmetry, LDF, laser speckle contrast imaging, LSCI), local blood pressure readings, or volume measurements (peripheral arterial tonometry, plethysmography) [2,11,12,13,14,15,16].

Flowmotion oscillations depend to some extent on the pulse (cardiac component, frequency around 1 Hz) and pulmonary pressure variations (respiratory-dependent component, 0.15–0.6 Hz). However, lower frequency oscillations in the microcirculation play a more important role. These can be subdivided into three groups: oscillations dependent on the vascular endothelium (endothelial component, ≤0.021 Hz), neurogenic activity (neurogenic component, 0.021–0.052 Hz), and smooth muscle activity (myogenic component, 0.052–0.15 Hz) [4,14,17,18,19,20,21,22,23].

Impaired flowmotion is associated with many diseases and disorders, including diabetes, hypertension, cardiovascular diseases, peripheral artery disease, chronic wounds, tumor progression, and erectile dysfunction [1,2,3,4,5,6,7,8,9,10]. In all these cases, a weakening of flowmotion is observed. For example, a large-scale Maastricht study (over 7200 participants) on flowmotion in diabetes type 2 (DM2) revealed a negative correlation between skin microvascular flowmotion and different measures of hyperglycemia. The decrease in flowmotion intensity applied to all low-frequency components of the oscillations [24].

Recently, we introduced Flow Mediated Skin Fluorescence (FMSF), a novel non-invasive diagnostic technique based on the measurement of nicotinamide adenine dinucleotide (NADH) fluorescence from skin tissue cells. The NADH/NAD^+^ redox state is an extremely sensitive internal marker for any changes in blood circulation and the delivery of oxygen to epidermal cells. The diagnostic potential of the method is based primarily on stimulation of the circulation in response to post-occlusive reactive hyperemia (PORH). The FMSF–PORH technique has been described in detail in numerous publications [25,26,27,28].

In contrast to changes in blood flow observed upon the full dilation and constriction of large arteries, microcirculation flowmotion is of intermediate intensity [3,29]. The NADH fluorescence signal during hyperemia/reperfusion changes by up to about 20–30%. This hyperemic response (HR_max_) is analogous to that in LDF and LSCI [15,30]. The changes during occlusion also reach about 15–25% of total fluorescence (unfortunately, other methods using PORH based on blood flow and local pressure changes remain “blind” during the occlusion period). The amplitude of the oscillations on the baseline (normoxia conditions) is on the order of 0.5% of the total fluorescence level and increases to 1–2% on the reperfusion line. Due to very low noise, the oscillations are clearly visible, including the cardiac component, which is several orders of magnitude weaker than the low-frequency oscillations. On the reperfusion line, the myogenic oscillations are so distinct that their frequency and amplitude can be determined without any complicated analysis [27,28].

In this study, we will focus only on myogenic oscillations and their role as an important regulator of microvascular adaptation to hypoxia. Myogenic vasomotion and the resulting myogenic flowmotion is controlled by Ca^2+^ oscillation in vascular smooth muscles, involving the periodic release of Ca^2+^ stores, oscillations in membrane depolarization, and elevated global cytosol Ca^2+^ concentrations [2,5,29,31]. Myogenic activity has been shown to be closely associated with effective oxygen delivery and increased hypoxia [32]. Increased myogenic oscillations have also been observed using LDF in mountain climbers ascending to high altitudes (above 3500 m). Increases in myogenic activity play a significant role in the adaptation of Sherpas highlanders to hypobaric hypoxia [33,34,35]. The activation of myogenic oscillations may therefore accompany hypoxic conditions present in numerous diseases, and could be used to assess microcirculatory functioning in diabetes, cardiovascular disease, peripheral arterial disease, and hypertension, as well as physical exercise tolerance [27]. Activation of vasomotion under hypoxic conditions also raises the question of how prevalent myogenic oscillations are under normal normoxic conditions, in both healthy and diseased individuals [2]. The predictive value of myogenic oscillation analysis under normoxic conditions to assess the microcirculatory response to hypoxia is discussed.

## 2. Materials and Methods

### 2.1. Brief Description of the Analyzed Groups

The analysis involved two main patient groups. One was a group of 910 patients with vascular diseases (CVD) and/or type 2 diabetes (DM2), as described in previous publications [10,28,36]. The second main group (control) consisted of healthy volunteers (77), athletes (76), amateur sportsmen (26) and endurance athletes (50), as described previously [27,37,38,39,40]. A third group of athletes consisted of 56 individuals (amateur sportsmen (26) and endurance athletes (30), average age 25.7 years), who were subjected to exertion to exhaustion. The final group comprised 9 competitive and amateur athletes (average age 26.2 years) given intermittent hypoxia treatment (IHT). The characteristics of the groups are displayed in Table 1.

All formal regulations concerning the collection of experimental data were respected and the patients were informed that the conducted measurements may be published under conditions of full confidentiality and anonymity.

### 2.2. The FMSF Methodology

**Figure 1:**
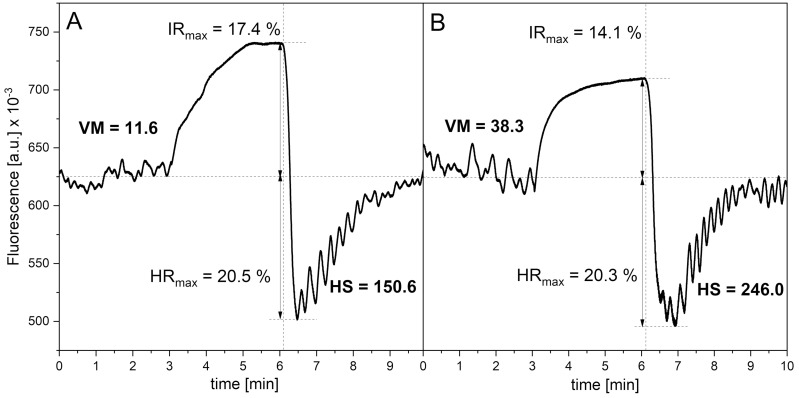
FMSF traces recorded for a physically active individual, male, age 40–45 y. before (**A**) and after (**B**) a single session of intermittent hypoxia treatment (duration 55 min, 5 min hypoxia at 9% O_2_ followed by 5 min normoxia at 21% O_2_).

The FMSF technique measures NADH fluorescence from the skin, mainly on the forearm. The methodological aspects of the FMSF method have been investigated and described in depth [25,26,27,28,37]. The diagnostic potential of the FMSF method is based primarily on stimulation of the circulation in response to post-occlusive reactive hyperemia (PORH) caused by the occlusion of a brachial artery, resulting in severe hypoxia in the limb downstream. Sudden release of the occluded artery causes reactive hyperemia, followed by a reperfusion period of a few minutes. Before and after occlusion, strong flowmotion oscillations are observed (Figure 1A). After normalization of the fluorescence signal and its correction for an unstable baseline, the share of each low-frequency oscillation components was determined using the Fast Fourier Transform (FFT) algorithm [27,41].

Under conditions of normoxia, vascular myogenic activity results in normoxic vascular myogenic oscillations (VM), which usually remain the weakest of the low-frequency oscillations. In contrast, oscillations after occlusion are dominated by the myogenic component and their intensity is referred to as hypoxia sensitivity (HS) (Figure 1). Both VM and HS are power spectra densities (PSD), calculated as the mean squared amplitude of myogenic oscillations under normoxia or hyperemia/reperfusion conditions, respectively, multiplied by a factor 10^6^. Whereas the VM and HS parameters vary within a very broad range, their logarithms remain normally distributed and can be used for statistical comparisons. Both numerical values are measured in arbitrary units.

### 2.3. Statistical Analysis

Statistical analyses were performed using OriginPro 2024 (10.1) software. The normality of the distribution was determined using the Shapiro–Wilk test. A two-sided *t*-test was used for the group comparison. Pearson’s correlation coefficient was calculated to assess the relationship between the continuous variables log(VM) and log(HS). The receiver operating characteristic (ROC) curve and the area under the curve (AUC) were calculated to set thresholds for the VM parameter. Sensitivity/specificity analysis was conducted to assess the predictive ability of the VM parameter to identify subjects with optimal, acceptable, and impaired adaptation of the microcirculation to hypoxia. A *p*-value of less than 0.05 was considered statistically significant.

## 3. Results and Discussion

The oscillatory changes in the NADH/NAD^+^ balance associated with hypoxia, and therefore the oscillations in NADH fluorescence monitored by the FMSF-PORH method, reflect microvascular vasomotion activated by occlusion of the brachial artery for 3 min. Myogenic oscillations are activated immediately following the occlusion, upon the release of blood flow. Their intensity (HS parameter) may be related to the instantaneous induction of HIF-1α in microvascular smooth muscle cells during transient hypoxia [42]. The better adaptation of microcirculation to hypoxia, the stronger activation of myogenic oscillations (higher values of HS). In the case of serious microcirculation disorders, myogenic oscillations remain very low and do not respond at all to hypoxic conditions, causing NADH/NAD^+^ imbalance.

**Figure 2:**
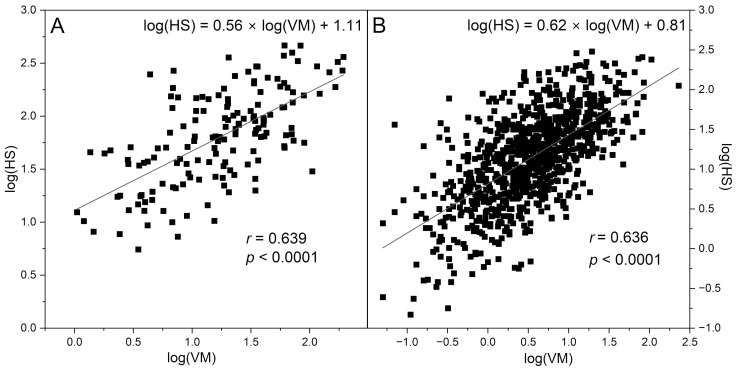
Linear correlation between the log(HS) and log(VM) parameters for the control group (**A**) and the CVD+DM2 group (**B**).

**Figure 3:**
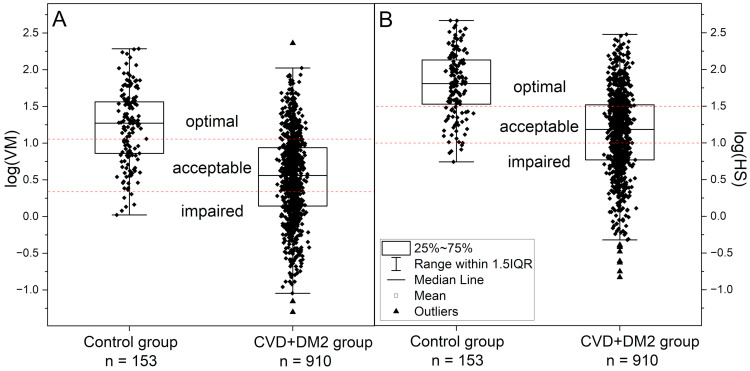
Distributions of the VM and HS parameters for myogenic oscillations (**A**) under normoxia conditions (log(VM)) and (**B**) in response to brachial artery occlusion (log(HS)) for the control group and patients with CVD+DM2 diseases.

An example of an FMSF-PORH course with spectacular activation of myogenic oscillations upon occlusion is presented in Figure 1A, for an active amateur athlete. Occlusion of the brachial artery for 3 min caused an over tenfold increase in the intensity of hypoxic myogenic oscillations HS (hyperemia/reperfusion period) vs. normoxic myogenic oscillations VM (baseline before the occlusion period) (see Figure 1A, VM = 11.6 (log(VM) = 1.06), HS = 150.6 (log(HS) = 2.18)). For the control group of 153 healthy volunteers and sportsmen, the myogenic oscillations under normoxia conditions log(VM) = 1.23 increased to log(HS) = 1.80 in response to transient hypoxia (see Table 1). The mutual relationship of log(HS) vs. log(VM) is well described by a linear relationship, with high Pearson’s correlation coefficients (see Figure 2A): log(HS) = (0.56 ± 0.06)∙log(VM) + (1.11 ± 0.07), *r* = 0.639, *p* < 0.0001. Both VM and HS parameters vary in a wide range of values (log(VM) = 0 ÷ 2.3, log(HS) = 0.7 ÷ 2.7; see Figure 3A,B).

The remarkable increase in myogenic activity is a response to hypoxia in the upper limb of the patient, caused by an occlusion (3 min) of the brachial artery in the PORH method. It is worth noting that such a long period of occlusion may cause some physical discomfort, anxiety, or even pain during measurement and so this methodology cannot be used for children or disabled persons.

As mentioned previously, reduced vasomotion often accompanies various diseases. For example, experimental results show significant differences in normoxic myogenic flowmotion between diabetic patients, prediabetic patients, and nondiabetic people [24]. Determining microvascular proper functioning and detecting dysfunction could play an important role in the pre-diagnosis of diabetic complications [4].

Recent studies using the Flow Mediated Skin Fluorescence (FMSF) technique performed for a group of 910 cardiovascular (CVD) and type-2 diabetic patients (DM2) have shown that the VM parameter, reflecting the intensity of myogenic microcirculatory oscillations under normoxia conditions (without occlusion of the brachial artery), correlates strongly with the HS parameter, which reflects the intensity of myogenic microcirculatory oscillations following hypoxia induced by occlusion of the brachial artery. Figure 2 shows the correlations of log(HS) vs. log(VM) observed for each group: A—control group, B—CVD+DM2 group. The linear dependence found for the CVD+DM2 group (see Figure 2B) of log(HS) = (0.62 ± 0.03)∙log(VM) + (0.81 ± 0.02), *r* = 0.636, *p* < 0.0001 remains similar to that found for the healthy control group.

The intensity of myogenic oscillations in these disease entities (CVD, DM2) ranges predominantly towards lower values of log(VM) = –1.3 ÷ 2.4 and log(HS) = –0.8 ÷ 2.5 in relation to the control group (see Figure 3A,B). Although both linear dependences in log(HS) vs. log(VM) have similar slopes, in the case of the healthy control, the same VM value corresponds to higher HS values than in the case of CVD+DM2 patients. In the control group, no individuals were observed with log(VM) < 0, and in the CVD+DM2 group there were no patients with log(HS) higher than 2.5.

The strong correlation between log(HS) and log(VM) allows the intensity of the response to hypoxia (log(HS)) to be predicted based on the assessment of normoxic myogenic oscillations (log(VM)) under normoxia conditions. Three ranges of HS have been proven to be indicative of different conditions of microvascular circulation, possessing diagnostic importance (HS < 10, log(HS) < 1, 10 ≤ HS < 30, and HS ≥ 30, log(HS) ≥ 1.48) [27]. Based on these three ranges for HS, diagnostic ranges for VM with similar meaning and importance can be selected: impaired VM (VM < 2, log(VM) < 0.30)), acceptable VM, (2 ≤ VM < 12), and optimal VM (VM ≥ 12, (log(VM) ≥ 1.08) (see Figure 3A,B).

The proposed partition of VM (log(VM)) into appropriate ranges does not significantly disturb the population of individuals diagnosed with impaired microvascular function, 32–38% in the CVD+DM2 patient group and only 3% in the control group, if we compare the partition of groups by log(VM) with respect to the same partition by log(HS).

Linking the relation of log(VM) with log(HS) via a linear relationship leaves some uncertainty regarding cases of false predictions of the proper response to hypoxia (HS) based on VM. Approximately 10% of all cases have log(VM) below 0.3, but at the same time log(HS) ≥ 1 (false negative group), while approx. 16% of all cases are those with log(VM) > 0.3, but at the same time log(HS) < 1 (false positive group). In part, this may result from measurement uncertainties. It is therefore advisable to repeat measurements for patients from impaired and acceptable groups. However, it may also be a real physiological effect. It is not an effect of age, since the log(VM) and log(HS) parameters for the group of cardiovascular and type 2 diabetes patients show a statistically significant dependence on age, and in both cases it is very similar (see Table 2). A stronger correlation of log(HS) and log(VM) with age was observed for females than for males in the CVD+DM2 group (Pearson coefficient around 0.3 for females, 0.2 for males), and both relationships have very similar slopes (log(HS) vs. age and log(VM) vs. age have slopes around −0.010 a.u./year for males, and around −0.014 a.u./year for females). The dependence on age is also similar to that found for the control group and remains in agreement with the literature and our previous observations [10,43].

**Figure 4:**
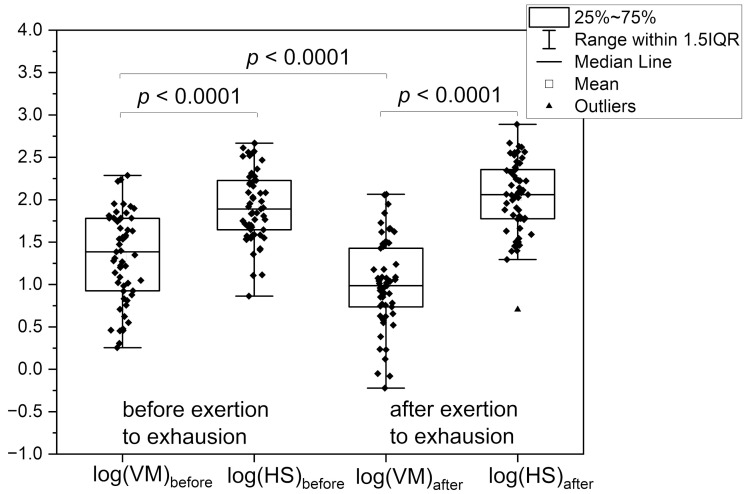
Changes in myogenic oscillations under normoxia conditions (log(VM)) and in response to brachial artery occlusion (log(HS)) before and after exertion to exhaustion for amateur and endurance athletes.

Possibly, this may be the result of chronic hypoxia or fatigue of the body due to a long-term disease, which in some patients translates into weakening or strengthening of myogenic oscillations under normoxia conditions, while maintaining the full potential of the hypoxia sensitivity (HS) response. Figure 4 shows such examples obtained for athletes and physically active individuals.

From the group of tested athletes, 26 amateur athletes and 30 endurance athletes exercised to exhaustion after the first FMSF-PORH measurement (Session 1: Exertion to exhaustion—before). The measurement performed again after this effort (Session 2: Exertion to exhaustion—after) indicated not only the almost complete disappearance of endothelial oscillations, as shown and discussed in our previous work [39], but also a significant decrease in myogenic oscillations VM for all athletes (session before exercise log(VM) = 1.32 vs. session after exercise log(VM) = 0.99, *p* < 0.001) (see Table 1, Figure 4). Despite the strong fatigue effect, the hypoxia sensitivity response (HS) remained almost unchanged (or even increased slightly but statistically insignificantly). Given the strong increase in myogenic oscillations in response to brachial artery occlusion (log(VM) vs. log(HS)), also presented in Figure 4, monitoring of changes in HS and VM can be considered as an important element of a well-planned all-day training cycle.

The opposite effect was observed as a result of a single session of intermittent hypoxia treatment, which is not such an extreme hypoxic event as exertion to exhaustion. As can be seen for the amateur sportsman and a group of a few volunteers (see Table 1), a single hypoxia treatment does not affected hypoxia sensitivity significantly: HS = 150.6 (log(HS) = 2.18) in Figure 1A vs. HS = 246 (log(HS) = 2.39) in Figure 1B. However, in this case, increased VM was observed after the intermittent hypoxia session: VM = 11.6 (log(VM) = 1.06) in Figure 1A vs. VM = 38.3 (log(VM) = 1.58) in Figure 1B. This increase is a residual effect of myogenic oscillations activation in response to hypoxia during the hypoxia session, as the second measurement was taken immediately after the treatment.

Both of the above-mentioned experiments clearly indicate strong stabilization of the HS response, which is a direct measure of the microvascular response to hypoxia. However, it should be remembered that hypoxia sensitivity (HS) may be affected by drugs or supplements which, through the increase in NO in vivo, transiently improve vascular circulation. It also shows some variability in the VM parameter, of approx. ±0.3 a.u. of log(VM) for athletes usually characterized by strong normoxic myogenic oscillations. Despite these uncertainties, the VM-based prediction of the myogenic oscillatory response to hypoxia seems an interesting alternative to measurements with brachial artery occlusion and assessment of the HS parameter, especially if more attention is paid to the results in the immediate vicinity of the threshold values.

**Figure 5:**
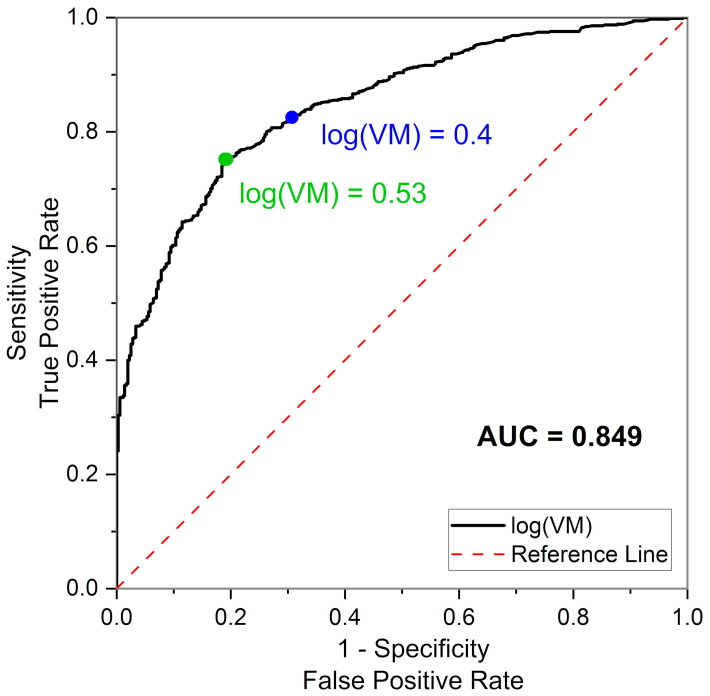
Receiver operating characteristic (ROC) curve obtained for all 1063 tested individuals (Control, CVD, DM2).

The selection of the threshold values of VM was subjected to more detailed analysis, especially the cut-off value for the impaired VM range. The receiver operating characteristic (ROC) curve was obtained for all 1063 tested individuals (Control, CVD, DM2), assuming a proper response to hypoxia as log(HS) ≥ 1 and impaired response as log(HS) < 1. The obtained AUC (area under curve) = 0.849 value confirms the predictive potential of VM. However, it can be clearly seen from the ROC curve presented in Figure 5 that the initially selected threshold value of log(VM) = 0.3 (VM = 2), although a highly sensitive predictor of the proper response to hypoxia (Sensitivity, TPR = 0.87), is not optimal in relation to the other ROC characteristics, especially Specificity (TNR = 0.58). The most optimal threshold value (maximizing Youden’s index) would be log(VM) = 0.53 (VM = 3.4, TPR = 0.75, FPR = 0.80).

Analysis of the ROC curve for the upper threshold, assuming an optimal response to hypoxia of log(HS) ≥ 1.48 (AUC = 0.81), indicates an optimal cut-off value for log(VM) = 0.83 (VM = 6.8, TPR = 0.67, FPR = 0.80).

The optimal cut-off points derived from ROC curves differ from the cut-off values derived from the log(HS) vs. log(VM) relationship. Moreover, they increase the size of the optimal group, and especially the impaired group (CVD+DM2), which is not justified by the fact that log(HS) is a more reliable measure of the microcirculatory response to hypoxia. It is worth noticing, however, that neither ROC curves possess clear inflection points and the change in Youden’s index is small over a wide range of log(VM).

A slight shift in the cut-off values within these wide maxima, the lower one to log(VM) = 0.4 (VM = 2.5, TPR = 0.83, FPR = 0.69) and the upper one to log(VM) = 0.9 (VM = 8, TPR = 0.62, FPR = 0.83), allows a similar percentage of indications to be obtained for the range of impaired, acceptable, and optimal responses to hypoxia for the CVD+DM2 patient group, using both log(HS) and log(VM).

As more results are obtained, the limits of the VM ranges can be adjusted accordingly.

**Figure 6:**
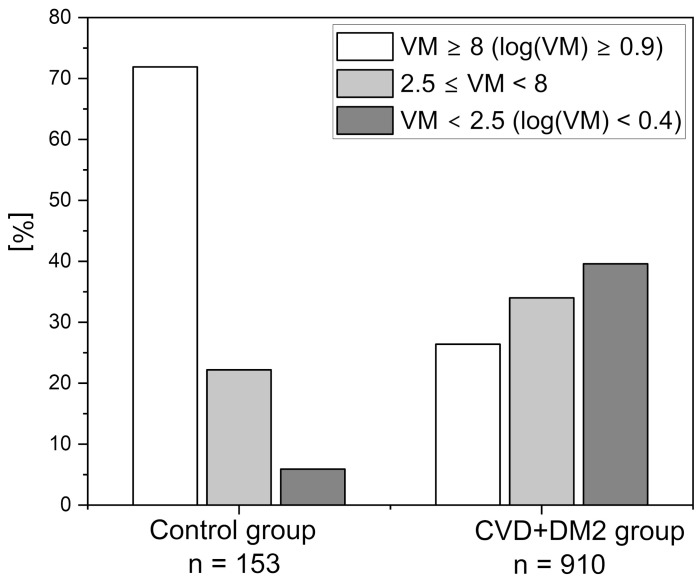
Distribution of patients according to the selected VM parameter values.

Figure 6 presents the distribution of the individuals tested according to the following ranges of VM: impaired log(VM) < 0.4, VM < 2.5, number of patients 351 (33%), acceptable 0.4 ≤ log(VM) < 0.9, 2.5 ≤ VM < 8, number of patients 343 (32%) and optimal log(VM) ≥ 0.9, VM ≥ 8, and number of patients 369 (35%) with partition into control and CVD+DM2 groups.

## 4. Conclusions

Although flowmotion analysis has been successfully used to assess microcirculation dysfunction in numerous disease entities, it has not yet become common clinical practice [2,4], mainly due to difficult measurement methodology, requiring a complicated analysis of results.

While many diseases are accompanied by a flowmotion disorder, or the disorder may even precede the main symptoms of the disease itself, there are numerous cases where the compensatory effect of myogenic microvasculatory adaptation to hypoxia can support the functioning of the body, as well as numerous examples of a complete lack of myogenic oscillations and their activation. Both conditions can be diagnosed either on the basis of a response to hypoxia, observed with the use of PORH provocation (log(HS) parameter), or on the basis of the predicted response from myogenic oscillations under normoxic conditions (log(VM) parameter). Both of these values remain strongly correlated with each other and similarly weaken with age. Optimal myogenic oscillations under normoxia conditions guarantee a good response to hypoxia under disease conditions. Their absence indicates a lack of support from microcirculatory flowmotion in combating a hypoxic condition. In the large group of cardiovascular and type 2 diabetic patients studied here, a significant proportion of them (one third) were deprived of such support. Similarly, decreased VM has been observed for patients with androgen deficiency [40], depression, and cancer (preliminary results).

If we consider HS as a measure of the microcirculation response to hypoxia, then VM can be considered a measure of microcirculatory readiness to provide this response. Thus, a simple, quick measurement without occlusion might have significant diagnostic value for health care workers. Such a less time-consuming measurement would be less harmful for the patient and more convenient for children and disabled people. It could be more easily introduced into the daily practice of athletes. Moreover, flowmotion assessment does not have to be limited to the upper limb, but can be performed on any part of the body using a movable measuring head.

## Figures and Tables

**Table 1:** Characteristics of the groups studied.

	Control Group	CVD+DM2 Group	Athletes Group		IHT Group	
			Before Exertion to Exhaustion	After Exertion to Exhaustion	Before Intermittent Hypoxia Treatment	After Intermittent Hypoxia Treatment
N	153	910 AH (508) CVD (475) DM2 (273)	56		9	
Female/Male	34/119	519/391	10/46		4/5	
Age [Years]	32.6 ± 11.7	67.6 ± 12.5	25.7 ± 4.8		26.2 ± 10.4	
BMI [kg/m^2^]	24.3 ± 4.0	28.9 ± 5.8	22.4 ± 2.4		20.6 ± 1.7	
DBP [mm Hg]	77.1 ± 10.3	74.9 ± 10.7	71.3 ± 7.7	72.9 ± 8.1	69.2 ± 7.8	72.2 ± 7.3
SBP [mm Hg]	130.4 ± 13.9	137.5 ± 18.1	129.7 ± 12.2	158.4 ± 19.8	118.3 ± 12.3	112.9 ± 7.8
IR_max_ [%]	16.3 ± 5.8	11.4 ± 5.6	18.6 ± 5.2	13.1 ± 5.4	20.1 ± 8.5	19.5 ± 4.1
HR_max_ [%]	19.8 ± 4.6	16.5 ± 5.1	20.6 ± 4.7	19.9 ± 4.8	18.5 ± 5.4	19.9 ± 5.6
log(VM)	1.23 ± 0.50	0.53 ± 0.58	1.32 ± 0.53	0.99 ± 0.52	1.06 ± 0.39	1.34 ± 0.34
log(HS)	1.80 ± 0.44	1.14 ± 0.56	1.91 ± 0.41	2.04 ± 0.43	1.89 ± 0.20	1.95 ± 0.22

Note: Continuous variables, mean ± SD. Abbreviations: AH, Arterial Hypertension; CVD, Cardiovascular Disease; DM, Diabetes Mellitus; BMI, Body Mass Index; DBP, Diastolic Blood Pressure; SBP, Systolic Blood Pressure; IR_max_, Ischemic Response maximal; HR_max_, Hyperemic Response maximal; log(VM), Vasomotion (logarithm); log(HS), Hypoxia Sensitivity (logarithm).

**Table 2:** Linear dependence of log(VM) and log(HS) on age.

	Control Group			CVD+DM2 Group		
	Slope [a.u./year]	*r* (Pearson Coefficient)	*p*-Value	Slope [a.u./year]	*r* (Pearson Coefficient)	*p*-Value
log(VM) vs. age	−0.017 ± 0.003	−0.385	<0.0001	−0.012 ± 0.002	−0.264	<0.0001
log(HS) vs. age	−0.012 ± 0.003	−0.311	<0.0001	−0.013 ± 0.002	−0.280	<0.0001

## Data Availability

Data sharing is not applicable. No new data were created in this study.
